# Modulation of Apoptotic Pathways by Human Papillomaviruses (HPV): Mechanisms and Implications for Therapy

**DOI:** 10.3390/v4123831

**Published:** 2012-12-18

**Authors:** Chung-Hsiang Yuan, Maria Filippova, Penelope Duerksen-Hughes

**Affiliations:** Department of Basic Sciences, Loma Linda University School of Medicine, 11085 Campus St., Loma Linda, CA 92354, USA; E-Mails: cyuan@llu.edu (C.-H.Y.); mfilippova@llu.edu (M.F.)

**Keywords:** apoptosis, HPV, papillomavirus, anti-HPV therapies, E2, E5, E6, E6*, E7

## Abstract

The ability of the host to trigger apoptosis in infected cells is perhaps the most powerful tool by which viruses can be cleared from the host organism. To avoid elimination by this mechanism, human papillomaviruses (HPV) have developed several mechanisms that enable the cells they infect to elude both extrinsic and intrinsic apoptosis. In this manuscript, we review the current literature regarding how HPV-infected cells avoid apoptosis and the molecular mechanisms involved in these events. In particular, we will discuss the modifications in intrinsic and extrinsic apoptotic pathways caused by proteins encoded by HPV early genes. Many of the current efforts regarding anti-cancer drug development are focused on directing tumor cells to undergo apoptosis. However, the ability of HPV-infected cells to resist apoptotic signals renders such therapies ineffective. Possible mechanisms for overcoming the resistance of HPV-infected tumor cells to anticancer drugs will be discussed.

## 1. Introduction

Human papillomaviruses (HPV) are small, double stranded DNA viruses that infect epithelial tissues, including those of the anogenital tract. Of the more than 100 different identified types of HPV, about 40 are involved in genital tract infections. Some types of HPV cause warts, while others can increase the risk of malignancy and lead to cancer of the cervix, other anogenital regions, and the head and neck areas [1]. HPV types are classified as either high risk or low risk, with high-risk types being associated with cancer formation while low risk types are not. For example, HPV types 6 and 11 are classified as low risk types, and infection with these types results in the proliferation of epithelial cells and manifests as warts or papillomas on the skin. However, these infections are generally self-limiting and do not lead to malignancy. On the other hand, HPV types 16 and 18 are major high-risk genotypes, which together cause up to 75% of cervical cancer cases as well as a significant number of head and neck squamous cell carcinomas. When high-risk HPV infects young females, most infections cause no or only minor symptoms, and usually disappear without any treatment within a few years. However, in 5% to 10% of infected women, the infection can persist for many years [2]. Persistent infections are at relatively high risk for causing cell abnormalities and developing lesions of the cervix, which can in turn lead to the development of cervical cancer. In 2010, 12,000 women in the United States were diagnosed with HPV-induced cervical cancer, of which 4000 were estimated to die. The development of HPV-induced cervical cancer usually requires 15–20 years, providing a window of opportunity for detecting the presence of HPV and/or HPV-induced cell abnormalities. Viral DNA can be detected before cell abnormalities are observed, and the Papanicolaou test, also called a Pap smear or Pap test, is used to screen and detect abnormal cells, possible precursors for malignancies. 

The HPV genome is divided into an early region (E), a late region (L) and a non-coding long control region (LCR). The E region encodes seven non-structural proteins: E1, E2, E4, E5, E6, E6* and E7, while the L region encodes two structural proteins: L1 and L2, which code for the major and minor capsid proteins, respectively. Early genes are responsible for modulating epithelial cell function so as to favor virus production. For example, their gene products modulate keratinocyte differentiation, promote viral DNA replication and segregation, and inhibit viral clearance by the immune system. All early HPV proteins perform multiple functions, and act by interacting with a variety of cellular partners [3,4]. These interactions in turn modify the flow through numerous cellular pathways, including apoptosis. The core proteins of the late genes, L1 and L2, together with HPV DNA, participate in the assembly of virus particles. During the normal viral life cycle, the HPV genome exists in host cells as an episome. In rare cases, however, integration of the viral genome into that of the host can occur. Interestingly, this integration is not required for the HPV life cycle and is, in fact, detrimental to the virus, as integrated viral sequences do not reproduce. However, integration is closely tied to the development of cancer, as most cases of HPV-induced cervical cancer feature an integrated form of the HPV genome. Such integration typically leads to an increase in the expression of HPV early proteins, including the E6 and E7 oncoproteins, and a consequential increase in cellular transformation and the probability of HPV induced carcinogenesis [5]. In this paper, we review the current literature regarding the molecular mechanisms through which HPV-infected cells avoid apoptosis. In particular, we will discuss the modifications in the intrinsic and extrinsic apoptotic pathways caused by proteins encoded by HPV early genes. This has clinical relevance, because many of the current efforts regarding anti-cancer drug development focus on directing tumor cells to undergo apoptosis.

## 2. Apoptosis

Apoptosis, also known as programmed cell death, plays an important role in development, cellular homeostasis and the pathophysiology of many diseases [6]. Apoptosis helps to eliminate damaged cells and also contributes to the elimination of virus-infected cells [7]. If inappropriately up- or down-regulated, apoptosis can also contribute to various diseases such as autoimmunity and cancer [8]. Two major classic apoptotic pathways have been identified: the extrinsic pathway, triggered by the engagement of “death receptors”, and the intrinsic pathway, which responds to a variety of stress signals (for example, radiation) by triggering the release of intracellular signals that promote cell death [9,10,11]. 

### 2.1 Extrinsic Pathway

Apoptosis is triggered through the extrinsic pathway when specific receptors are activated by their corresponding ligands. The majority of these “death” ligands belong to the TNF (tumor necrosis factor) family, and their corresponding cell surface “death” receptors to the TNF receptor family. TNF-alpha binds to TNF receptor 1 or 2 (TNF R1 and TNF R2), Fas ligand to Fas, and TRAIL (TNF-related apoptosis-inducing ligand) binds to Death Receptor (DR)1, DR2, DR3, DR4, and DR5. Other receptors within this family include FADD-like interleukin-1 beta-converting enzyme (FLICE) and TNF-like weak inducer of apoptosis (TWEAK) [12]. Adaptor molecules such as TRADD (Tumor necrosis factor receptor type 1-associated DEATH domain protein) and FADD (Fas-Associated protein with Death Domain) are engaged, and initiator caspases such as procaspase-8 are activated. Interactions of regions such as the death domain (DD) allow TRADD, FADD, and pro-caspase 8 to combine to form the death-inducing signaling complex (DISC) [13]. Formation of the DISC enables cleavage of caspase-8, which then cleaves and activates executioner caspases such as procaspase-3/7. The active caspase-3/7 can cleave many different target substrates, including poly (ADP-ribose) polymerase (PARP), and these cleavages lead to the demise of the cell. PARP is a nuclear enzyme that catalyzes the transfer of the ADP-ribose moiety of NAD+ to a specific subset of nuclear substrates in response to DNA damage. Active caspase-3 cleaves PARP into 24 kDa and 89 kDa segments, thereby eliminating the ability of PARP to function in DNA repair and thus promoting apoptosis. In the case of Fas-mediated apoptosis, two pathways have been shown to operate in a cell type-dependent manner (types I and II) [14]. In the type I cell model, a robust activation of caspase-8 can be observed following DISC formation. Overexpression of anti-apoptotic proteins such as Bcl-2 or Bcl-XL cannot stop the cleavage of caspase-8 and caspase-3, and mitochondrial activity is not required to trigger apoptosis in type I cells. In contrast, DISC formation in type II cells is weak, and additional signaling from the mitochondria is necessary for apoptosis. Low levels of DISC and capsase-8 are detected in type II cells. In this context, caspase-8 cleaves Bid and triggers mitochondrial depolarization and the release of cytochrome c. Following its release, cytochrome c forms a complex with apoptosis protease activating factor 1, (Apaf-1) and procaspase-9, called the apoptosome. The apoptosome is analogous in function to the DISC, and mediates the activation of caspase-9, which in turn activates caspase-3. The intracellular events of apoptosis then give way to the external characteristics of this form of cell death, which include chromatin condensation, phosphatidlyserine exposure, cytoplasmic shrinkage and membrane blebbing. Anti-apoptotic proteins such as Bcl-2 and Bcl-XL can block the activation of caspase in type II cells and significantly reduce apoptosis [14,15]. For ligands other than those of the TNF family, initiator caspases include capsase-10 and caspase-2, and the corresponding adaptor proteins are RIP, RAIDD/CRADD, and TRAFS. In most of these cases, activation of the executor caspases 3/7 initiates the apoptotic cascade [16,17,18,19]. 

### 2.2 Intrinsic Pathway

**Figure 1:**
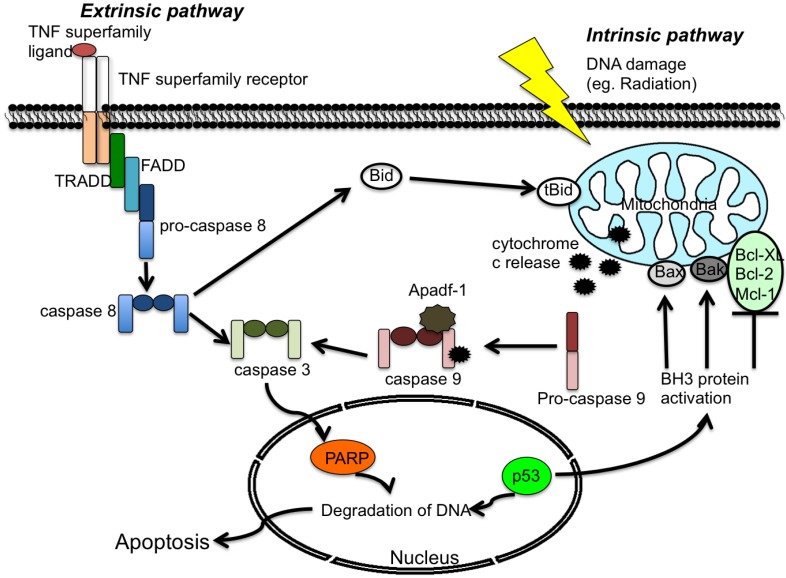
**Generalized overview of the extrinsic and intrinsic apoptosis pathways.** The extrinsic pathway is initiated by the binding of a tumor necrosis factor (TNF) family ligand to its receptor, followed by activation of downstream signaling proteins. In the intrinsic pathway, cellular stress causes BH3 activation and cytochrome c release. Both pathways activate the effector caspase-3 and promote cell death. Proteins are identified within the text.

The classic intrinsic apoptotic pathway, also referred to as the mitochondrial pathway, can respond to a variety of external stimuli such as DNA damage, radiation and osmotic stress [20,21]. Activation of this pathway results in the release of cytochrome c from the mitochondrial intermembrane space to the cytosol. Apoptotic protease activating factor-1 (Apaf-1), pro-caspase 9 and cytochrome c then form a complex called the apoptosome in the cytosol [22,23]. The binding of Apaf-1 and cytochrome c to procaspase-9 leads to the auto-cleavage and activation of procaspase-9. The active caspase-9 then cleaves and activates caspase-3, which induces the cleavage of additional substrates, such as PARP gelsolin, and protein kinase C-delta [24,25,26] (see Figure 1). 

## 3. Viruses and Apoptosis

Following infection of host cells, a virus typically employs a number of different mechanisms to avoid the host immune response and to initiate and maintain efficient production and spread of its progeny. In most cases, a host organism will attempt to initiate apoptosis in order to eliminate infected cells. For this reason, many viruses, especially the DNA viruses, encode a variety of viral proteins that can inhibit or delay these protective actions, often by targeting key cellular proteins [11]. For example, human adenovirus employs the E3 14.7 kDa protein to reduce TNF- and Fas-induced apoptosis; these proteins work by inhibiting the TNF R-induced release of arachidonic acid [27]. Poxvirus produces two proteins, CrmB and CrmC, that change the conformation of TNF R in order to block the binding of TNF [28]. In addition, poxvirus and herpesvirus both encode proteins with homology to the DED domain, and thus block the binding of death receptors and the FADD adaptor protein to caspase-8 [29]. CD40 and CD40L form a receptor/ligand pair found on antigen presenting and T cells, respectively, and is required for their activation. Epstein-Barr virus (EBV), a causal agent of epithelial and lymph tumors, can increase the expression of CD40L but down-regulates the expression of CD40. This abnormal interaction between CD40L and CD40 contributes to decreased apoptosis of infected cells and the development of myeloma cells [30]. Furthermore, the Epstein-Barr nuclear antigen 3C (EBNA3C) is known to possess anti-apoptotic properties and to regulate cell proliferation. For example, EBNA3C can bind to p53 and Gemin3 so as to inhibit the binding between p53 and DNA, thus blocking the p53-mediated apoptotic pathway [31]. HPV also expresses several proteins that affect cellular apoptotic pathways, as detailed below.

## 4. The HPV Life Cycle

HPV infects cells in the basal layer, below the surface of the epithelium, and carries out a life cycle that is closely tied to the differentiation program of the host cells. During the early stages of infection, the episomal viral genome is usually present at fewer than 100 copies per cell, and functions as a slowly replicating plasmid. Shortly after infection and uncoating, low-level expression of early genes (E6, E6*, E7, E1 and E2) as directed by the early promoter, enables maintenance of the episomal state of the HPV genome [32,33,34,35,36].

As cells move up through the layers, they begin to proliferate. The ability of E6 and E7 to suppress apoptosis and to alter the function of factors involved in cell-cycle regulation, respectively, facilitates prolongation of the proliferative stage of keratinocyte differentiation [37,38]. During this proliferative stage, E1 and E2 function to regulate viral DNA replication and transcription such that viral DNA is replicated coordinately with the host cell chromosome. E2 binds to the HPV upstream regulatory region and recruits the E1 DNA helicase to the viral origin of replication. In addition, E2 acts to bind the necessary factors onto mitotic chromatin and to tether the virus genome to host chromosomes during mitosis [39]. Meanwhile, E1 binds to the viral origin of replication. It then uses ATP to unwind double-stranded DNA, acting like a helicase. 

Amplification of viral genomes begins in the proliferative layers, and an increased level of expression of all the early viral gene products, including E4 and E5 [40,41,42], is necessary for this process. During this stage, an increase in transcription of the genes coding for the E1, E2, and E1-E4 fusion proteins is observed [43,44]. The sequence of E4 is located entirely within the E2 ORF, and the protein is expressed relative late during viral infection. E2 is co-expressed with and binds to E1^E4 protein and this interaction results in the stabilization of both proteins [45]. Overexpression of the E1^E4 protein enables reorganization of keratin network during keratinocyte differentiation and viral genome amplification [46].

Both the E4 and E5 proteins participate in the late viral functions, although details of these mechanisms are not yet entirely defined. It has been shown that E5 plays a subtle role during the productive stage of the HPV16 life cycle [41,47]. When the infected cells reach the point of terminal differentiation, the viral copy number is increased up to a thousand per cell, and the late genes L1 and L2, which encode viral capsid proteins, are expressed. The viral capsid proteins assemble into virus particles and are released and spread to other host cells. Through these mechanisms, HPV is able to enhance the survival of infected cells in order to facilitate its own replication cycle, and thus ensure the production and spread of progeny. 

## 5. HPV Proteins that Modulate Apoptosis

Apoptosis is an important mechanism through which the host can eliminate infected cells, and the ability to avoid apoptosis can therefore enhance virus survival. In addition, apoptosis plays an important role during keratinocyte differentiation [48]. For these reasons, it is to the virus’ advantage to intervene in apoptotic pathways, and five papillomavirus proteins, E2, E5, E6, E6* and E7, have been shown to do so. E5 and E6 can protect host cells from apoptosis, while E2 and E6* appear to possess pro-apoptotic properties. E7 can act in either a pro-apoptotic or an anti-apoptotic manner, depending on the cell type and circumstances. While it is not entirely clear why pro-apoptotic properties of virus proteins might enhance virus survival and propagation, it is likely that the influence of each HPV protein on host apoptotic pathways is related to specific stages of the virus life cycle. Overexpression of the major oncogenes, E6 and E7, during cell transformation very often results in resistance of transformed cells to apoptosis; this ability to avoid apoptosis is one of the major hallmarks of carcinogenesis [49]. 

### 5.1. HPV E2

E2 functions as a viral transcription factor, and also plays a role in HPV expression and viral replication. It has a transcriptional activation domain near its N-terminus and a DNA binding domain near the C-terminal end. These two regions are separated by a flexible hinge [50]. In earlier studies, over-expression of HPV E2 in HPV-transformed cells was able to reduce E6 and E7 expression and thus to induce apoptosis. This experiment involved transfection of the BPV or HPV-18 E2 gene into HeLa cells, which resulted in an increased level of apoptosis [51]. E2 can also induce apoptosis in cells that do not contain the HPV genome, indicating that the pro-apoptotic activity of E2 can also work independently of HPV viral function and cell type. This type of E2-induced apoptosis proceeds through the extrinsic pathway, with the activation of caspase-8 [52]. E2 does this by binding to the caspase-8 DED domain and thus initiating activation. There are two structural characteristics of E2 known to be involved in apoptosis. First, the N-terminal of HPV-18 E2 contains a 27 amino acid α-helix, which can induce caspase oligomerization and cell death [53]. Second, the HPV-18 E2 protein includes a transactivation domain, also near its N-terminus, which can induce apoptosis *via* p53-independent mechanisms [52]. In addition, the HPV E2 protein binds to p53 [54], and has the ability to induce apoptosis through the p53-dependent pathway in both HPV-transformed cells and normal cells [55]. Interestingly, the mutated E2 protein, VP22-E2, which carries a mutation within the p53-binding site, can induce apoptosis in HPV-transformed cells, but has less of an effect in normal cells [56]. Other reports have demonstrated that HPV-16 E2 loses its ability to induce apoptosis if the N-terminal domains are removed. In contrast, mutations that block the DNA binding domain of E2 have no effect on its ability to induce apoptosis [55]. 

It should be noted that these pro-apoptotic functions of E2 were demonstrated in experiments utilizing transient over-expression, and that the levels of E2 resulting from this approach are much higher than would be found under more physiological conditions. During the HPV life cycle, expression of E2 is typically quite low in keratinocytes at early stages of differentiation, and may not be sufficient to induce apoptosis. In later stages when E2 expression is higher, the production of E4 may reduce the pro-apoptotic activity of E2. Alternatively or in addition, this pro-apoptotic activity of E2 may coincide with apoptosis induced during normal keratinocyte differentiation. During carcinogenesis, often triggered by HPV integration, the activity of E2 is likely to drop because integration frequently disrupts the E2 region. 

### 5.2. HPV E5

HPV E5 is considered an oncogene because it can induce transformation in murine fibroblasts and human keratinocytes [57]. In addition, E5 can induce skin carcinogenesis in transgenic mice [58]. E5 employs multiple mechanisms with the potential to contribute to malignant transformation. For example, several studies show that HPV-16 E5 can interact with the epidermal growth factor receptor (EGFR) signaling pathway [57,59,60]. Over-expression of EGFR signaling plays an important role in the development of many cancers, as the activation of EGFR can regulate gene transcription and modulates cell proliferation, apoptosis, angiogenesis, tumor invasion and metastasis through the Ras-Raf-MAP kinase pathway and/or the PI3K-Art pathway [61]. In addition, E5 expression can down-regulate expression of MHC/HLA class I, thus facilitating evasion of the host immune response [47]. With regards to the regulation of apoptosis, it is known that HPV-mediated cervical cancers express decreased levels of Fas, which leads to impaired apoptosis. E5 may contribute to this, as HPV 16 E5 has been shown to reduce FasL and TRAIL-mediated apoptosis by down-regulating Fas expression and altering the formation of the death-inducing signaling complex [62]. It should be noted that these experiments employed over-expression of E5 in HaCaT cells. HPV-16 E5 can also protect cells from UV-induced apoptosis by enhancing the PI3K-Art and MAP kinase pathways [63]. Finally, the E5 protein can inhibit hydrogen peroxide-induced apoptosis by stimulating ubiquitin and proteasome-mediated degradation of Bax, a pro-apoptotic protein [64]. Through such mechanisms, therefore, the E5 protein can prevent HPV-infected cells from responding to apoptotic stimulation [65]. 

### 5.3. HPV E6

HPV E6 is an essential oncoprotein that has been widely studied over the last two to three decades. E6 is a small protein, consisting of 151 amino acids and presenting two atypical zinc fingers with motifs that contain two cysteines (Cys-X-X-Cys). The p53 tumor suppressor is the first-described and best-known target of HPV E6 [66]. p53 acts as a transcriptional factor, and can trigger cell cycle arrest or apoptosis in response to cellular stress or DNA damage. Under normal conditions, triggers such as DNA damage cause increases in the level of p53, which then lead to downstream effects such as cell cycle arrest and/or apoptosis, depending on the intensity or amplitude of the damage or stimulus. The presence of E6 from high-risk types of HPV interferes with this process, because E6 binds to both p53 and E6-associated protein ligase (E6AP), causing ubiquitinylation and the subsequent degradation of p53. This loss then prevents p53 from inducing either growth arrest or apoptosis of infected cells [67]. During this process, E6 first binds to E6AP, which functions as an E3 ubiquitin protein ligase. Then, the E6/E6AP complex binds to the core domain of p53. E6AP catalyzes the transfer of ubiquitin to p53, thereby enabling its proteasome-mediated degradation. This E6-mediated inhibition of p53 activity can increase the survival of transformed cells [68,69]. Interestingly, a recent study showed that E6 could also interact with ubiquitin ligases other than E6AP in order to promote p53 degradation [70]. 

In addition to p53, E6 also interacts with other partner proteins that play a variety of roles in the cell [3,71]. Following binding to E6, many of these proteins are lost by an E6AP-mediated ubiquitinylation and degradation process similar to that observed for p53. E6 partners include proteins involved in apoptosis such as Bak [72], c-Myc [73], TNF R1 [74], FADD, and caspase-8 [3,75,76,77]. Bak and Myc were the first apoptosis-related proteins, other than p53, to be identified as targets of E6. Following binding to E6, both Bak and Myc are degraded through the ubiquitin-proteasome pathway [73]. This loss of Bak has biological consequences, as E6 has been shown to inhibit TNF-mediated apoptosis by reducing the expression of Bak in a process independent of the regulation of caspase-3 and caspase-8 expression [78]. Other key proteins involved in cellular apoptosis can also be compromised by E6. For example, E6 can bind to the death effector domains (DEDs) of FADD and procaspase-8 and accelerate their degradation [75,76,77,79]. The resulting lower amounts of FADD and procaspase 8 in E6-expressing cells then hinders formation of the apoptotic Death Inducing Signaling Complex (DISC) that would normally be triggered by members of the TNF superfamily, thereby compromising the ability of TNF, FasL and TRAIL to initiate apoptosis [74,75,77,80]. In addition, E6 exerts effects on other apoptotic and anti-apoptotic proteins at the transcriptional level. For example, E6 can up-regulate the activity of the survivin promoter [81]. 

In each of these cases, E6 functions to reduce cell death by compromising the integrity of cellular pathways leading to apoptosis. In the context of the virus life cycle, the prevention of apoptosis is critical because it protects infected cells from elimination by the immune system, and therefore enables them to continue serving as hosts. This ability may also prolong the early stages of the life cycle. During carcinogenesis, the expression of E6 is usually increased, resulting in cells that do not undergo apoptosis and that are then susceptible to E6 and E7-mediated transformation. One recent study found that E6-mediated cervical cancer relies on the continuous expression of E7 along with constitutively expressed E6, and that continuous expression of E7 is required for the maintenance of cervical cancer and precancerous lesions in transgenic mice [82].

### 5.4. HPV E6*

E6*, a splice isoform corresponding to approximately the N-terminal half of E6, has significance in both the viral life cycle and in carcinogenesis. Early work suggested that the purpose of the splicing events that produce the HPV16 E6 splice variants was to facilitate translation of E7 [83]. However, more recent research has called this into question [84], and several reports now demonstrate significant and independent roles for this splice variant [79,85,86,87]. For example, over-expression of E6* in SiHa cells was shown to sensitize those cells to TNF-and Fas-induced apoptosis [76]. Furthermore, it was shown that while the full-length E6 and E6* proteins both bind to procaspase-8, they affect its stability in opposite directions. That is, while binding of the full-length isoform to procaspase 8 leads to instability and an acceleration of degradation, binding of the short isoform, E6*, leads to procaspase 8 stabilization and an increase in activity [79]. Interestingly, the full-length and short versions of E6 bind to different regions of procaspase 8 [79]; this is likely linked to the different consequences of this binding. Results such as these indicate that E6* performs different functions than does the full-length isoform, and that E6* may function in a pro-apoptotic manner. It may be that the pro-apoptotic characteristics of E6* reflect a general function of E6* as a regulator of the potent E6 oncogene. 

### 5.5. HPV E7

HPV E7 is also a well-known oncoprotein with multiple functions, including the inhibition of differentiation and the activation of cell cycle progression. E7 acts by releasing active E2F, thereby enabling transcription of several genes involved in DNA synthesis [88]. The primary target of E7 is the retinoblastoma protein (pRb), along with related proteins such as p107 and p130 [89]. These proteins bind to the E2F transcription factor, and are involved in regulation of the cell cycle, and in particular, regulation of the transition from G1 to S phase. Normally, in the absence of growth-promoting signals, the binding of pRb to E2F prevents E2F from activating its target genes, which are necessary for entrance into S phase and thus for cellular proliferation. When growth-promoting signals are present, however, phosphorylation of pRb, mediated by cyclin-dependent kinase (CDK) activity, leads to disruption of pRb/E2F complexes and the release of E2F. E2F is thus activated and promotes the re-entrance of cells into S phase. The HPV E7 protein performs a function analogous to pRb phosphorylation, in that the virus protein binds to pRb and releases E2F. This results in the activation of E2F-responsive genes, which encode proteins essential for both cell cycle progression and virus replication. In addition to its role in regulating the cell cycle, HPV-16 E7 can also induce cells to undergo apoptosis [90,91]. For example, HPV-16 E7 could induce apoptosis in the retina from transgenic mice expressing this viral oncogene in a p53-dependent manner [92]. In cell models, E7 from both low-risk (HPV-1, HPV-60) and high-risk HPVs (HPV-16) was shown to induce apoptosis in immortalized rodent fibroblasts (NIH3T3 cells) [93], and the co-expression of E7 and p21 proteins induced apoptosis in U2OS cells [94]. However, it should be noted that these pro-apoptotic effects of E7 were demonstrated in the context of models that are not natural targets for HPV. In a more relevant model, over-expression of E7 in human primary keratinocytes induced spontaneous cell death and also sensitized the cells to TNF-mediated apoptosis [95]. Interestingly, however, other research indicates that E7 can inhibit apoptosis in some cell types. For example, HPV-16 E7 inhibits Fas- and TNF-mediated apoptosis through the suppression of caspase-8 activation in human fibroblasts [96], and the expression of E7 in HaCaT cells reduced apoptosis following the application of genotoxic stressors such as the alkylating agent mitomycin C or UV [97]. The mechanism(s) for these effects are still not clear; however, a recent study showed that HPV-16 E7 can interact with the pro-apoptotic cellular factor, Siva-1, and inhibit UV-induced apoptosis in HaCaT cells [98]. Together, these studies demonstrate that E7 can both induce and inhibit apoptosis, depending on the cell and virus types. Any pro-apoptotic functions displayed by E7 are likely to be relatively weak, at least as compared to the anti-apoptotic functions displayed by E6, because in experiments in which both E6 and E7 are expressed, target cells become resistant to apoptosis induced by several stimuli [99,100].

## 6. Re-Engagement of Apoptotic Pathways as a Therapeutic Approach to HPV-Induced Cancers

The association between HPV and human cancer has been studied for more than three decades. HPV-induced cervical cancer is the second most common cancer and the fifth leading cause of cancer-related deaths among women worldwide [101,102]. Cervical cancer is not the only cancer caused by high-risk types of HPV; in fact, biological and functional studies have demonstrated the role of HPV in the development of several additional types of cancer, such as cancer of the head and neck, vaginal cancer, vulvar cancer, anal cancer, and penile cancer [103,104]. 

Recently, two HPV vaccines, Gardasil (MSD) and Cervarix (GSK), have been developed and approved for the prevention of infection with either two or four types of HPV. These vaccines are therefore predicted to reduce the incidence of cervical cancer. In Phase III randomized clinical trials, for example, Cervarix was able to prevent 90% of moderate and severe precancerous cervical lesions associated with HPV-16 and 18 [105]. However, while these vaccines can prevent HPV infection, they offer no benefit to an individual who has already been infected with HPV. Also, the vaccines will not have a significant effect on human health for decades, because most women are infected in their late teens/early twenties, while cancer appears in their late forties/early fifties. Furthermore, the vaccines only protect against HPV-16 and -18, leaving 25–30% of high-risk infections unaffected by an individual’s vaccination status. An additional issue is the high cost of these vaccines, making it difficult to deliver them to areas and populations with low resources. 

Surgical treatment is often employed to cure cervical cancer in the early stages, but it is not always easy to use this approach in head and neck cancers or in laryngeal papillomatosis, due to the need to maintain the normal structure of the airway and to avoid pulmonary spread. One option currently used to treat recurrent respiratory papillomatosis is endolaryneal laser surgery. Following surgical excision, however, HPV-associated cancers frequently return, especially in the immunocompromised population. Combined chemoradiotherapies have been assessed, with variable results [106]. Topical treatments, such as trichloroacetic acid, liquid nitrogen, imiquimod, interferon-α injections and podophyllin resin, are also available [107,108,109,110]. However, repeated treatments are necessary and are not always effective. Furthermore, imiquimod has not been approved for use in the oral cavity. Even when a wart or papilloma can be removed by such approaches, the HPV infection has not necessarily been cured and may return. In conclusion, there is no effective antiviral agent currently available. 

To expand and enhance the limited options currently available, several laboratories have begun examining the possibility of using molecular strategies to inhibit HPV [111,112]. For example, the HPV E1 and E2 proteins are involved in replication and gene expression, and could, in theory, be targeted by small molecules. Replication of the HPV genome requires the viral helicase E1 and the origin-binding protein E2, and is initiated by the binding of E1, E2 and specific DNA sequences within the viral origin of replication. As one example, biphenylsulfonacetic acid has been shown to inhibit E1 ATPase activity, and the optimized compound has an IC50 value of 4.0 nM [113]. Unfortunately, the expression of these two viral proteins is often lost during the process of malignant transformation, making them less-than-optimal targets for cancer treatment. In contrast, the expression of HPV E6 and E7 usually increases during this stage, and for this reason, these two oncoproteins have the potential to serve as useful targets for small molecule inhibitors. For example, the prevention of E6-mediated p53 degradation, perhaps by inhibiting formation of the E6/E6AP complex, or preventing the interaction between E7 and Rb, represent promising approaches for the development of small molecule inhibitors of HPV-associated cancer [114].

Currently, therapeutic approaches that rely on activation of either the intrinsic or the extrinsic apoptotic pathways are unlikely to be helpful for HPV-associated diseases due to the ability of high-risk E6 proteins to subvert both extrinsic and intrinsic apoptotic pathways by mediating the rapid degradation of signaling molecules (p53 in the case of the intrinsic pathway and FADD and caspase 8 in the case of the extrinsic pathway). As a result of these E6 activities, therefore, engagement of either the extrinsic or the intrinsic apoptotic pathways does not result in the transduction of the intended death signal because the mediator molecules are missing. Therefore, if any of these apoptosis-inducing signaling pathways are to be used as tools for the elimination of HPV-associated malignancies, it will be necessary to restore the missing signaling molecules.

An approach based on inhibiting the ability of E6 to mediate the rapid degradation of its cellular partners has the potential to re-sensitize HPV^+^ cells to inducers of apoptosis, and could therefore make certain existing cancer treatments available to those suffering from HPV-associated malignancies. Progress has been made in the search for inhibitors of the E6/E6AP interaction; this approach has the potential to re-activate the intrinsic apoptotic pathway. First, the essential binding motif through which E6 and E6AP interact was identified and verified [42,115,116]. Structural information regarding the E6/E6AP binding motif was then transferred to a three-dimensional query format suitable for computational screening of large chemical databases. This 3D format was used to query the National Cancer Institute (NCI) open chemical database comprising approximately 240,000 compounds, as well as the Sigma-Aldrich Library of Rare Chemicals of 97,000 compounds. Selected molecules that were predicted to fit into the E6/E6AP binding motif were then analyzed for their ability to inhibit E6/E6AP interactions and thereby interfere with E6-promoted p53 degradation. Following these *in vitro* and cell-based assays, several compounds with these characteristics were identified; such compounds have the potential for development into new therapeutic agents for treatment of HPV-associated cancers [117].

It may also be possible to target the extrinsic apoptotic pathway. For example, activation of the TRAIL-mediated, extrinsic apoptotic pathway shows promise in the treatment of several types of tumors. TRAIL-based therapies have elicited significant interest in recent years, largely due to their apparent ability to kill tumor cells while sparing most normal cells. Previous studies have reported on the effectiveness of both TRAIL and α-TRAIL R1 and R2 in combination with other sensitizing agents such as doxorubicin, bortezomib, adriamcin, 5-fluorouracil, irinotecan hydrochloride, paclitaxel, carboplatin, gemcitabine, cisplatin and radiation in both cell and animal model systems [118,119,120,121]. Work from our laboratory has shown that a peptide corresponding to the E6-binding site can block the interaction between HPV E6 and FADD, thus re-sensitizing SiHa cells to TRAIL- and Fas-induced apoptosis [79]. Small molecules represent another approach, as they have been used to inhibit important signal transduction pathways involved in breast, colon, pancreatic and lung cancer formation [122]. Once small molecules block the inhibitory activity of E6, known triggers of apoptosis, such as TRAIL, could initiate apoptosis of HPV-positive cells. We recently screened a small molecule library and found several chemical compounds that had the ability to inhibit the interaction between E6 and procaspase-8 [123]; a subset of these molecules can re-sensitize SiHa cells to TRAIL (data not shown). Such findings suggest that targeting HPV E6 or other oncoproteins could prove an excellent therapeutic strategy to treat HPV-associated cancers, and that small molecules could prove an appropriate approach to reach this goal.

## References

[1] Stanley M.A. (2012). Genital human papillomavirus infections: current and prospective therapies. J. Gen. Virol.

[2] Goldstein M.A., Goodman A., del Carmen M.G., Wilbur D.C. (2009). Case records of the Massachusetts General Hospital. Case 10–2009. A 23-year-old woman with an abnormal Papanicolaou smear. N. Engl. J. Med..

[3] Tungteakkhun S.S., Filippova M., Neidigh J.W., Fodor N., Duerksen-Hughes P.J. (2008). The interaction between human papillomavirus type 16 and FADD is mediated by a novel E6 binding domain. J. Virol..

[4] Moody C.A., Laimins L.A. (2010). Human papillomavirus oncoproteins: Pathways to transformation. Nat. Rev. Cancer.

[5] Grm H.S., Massimi P., Gammoh N., Banks L. (2005). Crosstalk between the human papillomavirus E2 transcriptional activator and the E6 oncoprotein. Oncogene.

[6] Rudin C.M., Thompson C.B. (1997). Apoptosis and disease: regulation and clinical relevance of programmed cell death. Annu. Rev. Med..

[7] Mundt B., Kuhnel F., Zender L., Paul Y., Tillmann H., Trautwein C., Manns M.P., Kubicka S. (2003). Involvement of TRAIL and its receptors in viral hepatitis. Faseb. J..

[8] White S., Rosen A. (2003). Apoptosis in systemic lupus erythematosus. Curr Opin Rheumatol.

[9] Chen W., Sulcove J., Frank I., Jaffer S., Ozdener H., Kolson D.L. (2002). Development of a human neuronal cell model for human immunodeficiency virus (HIV)-infected macrophage-induced neurotoxicity: apoptosis induced by HIV type 1 primary isolates and evidence for involvement of the Bcl-2/Bcl-xL-sensitive intrinsic apoptosis pathway. J. Virol..

[10] Fulda S., Debatin K.M. (2006). Extrinsic versus intrinsic apoptosis pathways in anticancer chemotherapy. Oncogene.

[11] Taylor J.M., Barry M. (2006). Near death experiences: poxvirus regulation of apoptotic death. Virology.

[12] Gaur U., Aggarwal B.B. (2003). Regulation of proliferation, survival and apoptosis by members of the TNF superfamily. Biochem. Pharmacol..

[13] Ashkenazi A., Holland P., Eckhardt S.G. (2008). Ligand-based targeting of apoptosis in cancer: The potential of recombinant human apoptosis ligand 2/Tumor necrosis factor-related apoptosis-inducing ligand (rhApo2L/TRAIL). J. Clin. Oncol..

[14] Scaffidi C., Fulda S., Srinivasan A., Friesen C., Li F., Tomaselli K.J., Debatin K.M., Krammer P.H., Peter M.E. (1998). Two CD95 (APO-1/Fas) signaling pathways. Embo J..

[15] Schmitz I., Walczak H., Krammer P.H., Peter M.E. (1999). Differences between CD95 type I and II cells detected with the CD95 ligand. Cell. Death Differ..

[16] Wang J., Chun H.J., Wong W., Spencer D.M., Lenardo M.J. (2001). Caspase-10 is an initiator caspase in death receptor signaling. Proc. Natl. Acad. Sci. USA.

[17] Tinel A., Tschopp J. (2004). The PIDDosome, a protein complex implicated in activation of caspase-2 in response to genotoxic stress. Science.

[18] Henshall D.C., Skradski S.L., Bonislawski D.P., Lan J.Q., Simon R.P. (2001). Caspase-2 activation is redundant during seizure-induced neuronal death. J. Neurochem..

[19] Sohn D., Budach W., Janicke R.U. (2011). Caspase-2 is required for DNA damage-induced expression of the CDK inhibitor p21(WAF1/CIP1). Cell. Death Differ..

[20] Rich T., Allen R.L., Wyllie A.H. (2000). Defying death after DNA damage. Nature.

[21] Vermeulen K., Van Bockstaele D.R., Berneman Z.N. (2005). Apoptosis: Mechanisms and relevance in cancer. Ann. Hematol..

[22] Li P., Nijhawan D., Budihardjo I., Srinivasula S.M., Ahmad M., Alnemri E.S., Wang X. (1997). Cytochrome c and dATP-dependent formation of Apaf-1/caspase-9 complex initiates an apoptotic protease cascade. Cell..

[23] Zou H., Li Y., Liu X., Wang X. (1999). An APAF-1.cytochrome c multimeric complex is a functional apoptosome that activates procaspase-9. J. Biol Chem.

[24] Hill M.M., Adrain C., Duriez P.J., Creagh E.M., Martin S.J. (2004). Analysis of the composition, assembly kinetics and activity of native Apaf-1 apoptosomes. Embo J..

[25] Kothakota S., Azuma T., Reinhard C., Klippel A., Tang J., Chu K., McGarry T.J., Kirschner M.W., Koths K., Kwiatkowski D.J. (1997). Caspase-3-generated fragment of gelsolin: Effector of morphological change in apoptosis. Science.

[26] Kato K., Yamanouchi D., Esbona K., Kamiya K., Zhang F., Kent K.C., Liu B. (2009). Caspase-mediated protein kinase C-delta cleavage is necessary for apoptosis of vascular smooth muscle cells. Am. J. Physiol. Heart Circ. Physiol..

[27] Zilli D., Voelkel-Johnson C., Skinner T., Laster S.M. (1992). The adenovirus E3 region 14.7 kDa protein, heat and sodium arsenite inhibit the TNF-induced release of arachidonic acid. Biochem. Biophys. Res. Commun..

[28] Rahman M.M., Lucas A.R., McFadden G. (2009). Viral TNF inhibitors as potential therapeutics. Adv. Exp. Med. Biol..

[29] Bertin J., Armstrong R.C., Ottilie S., Martin D.A., Wang Y., Banks S., Wang G.H., Senkevich T.G., Alnemri E.S., Moss B., Lenardo (1997). Death effector domain-containing herpesvirus and poxvirus proteins inhibit both Fas- and TNFR1-induced apoptosis. Proc. Natl. Acad. Sci. USA.

[30] Zhang Y., Zhao H., He X., Zheng S., Wang T., Yan D., Sun J., Lu X., Wen J., Lau W.Y. (2012). Effects of Epstein-Barr virus infection on the development of multiple myeloma after liver transplantation. Sci. China Life Sci..

[31] Cai Q., Guo Y., Xiao B., Banerjee S., Saha A., Lu J., Glisovic T., Robertson E.S. (2011). Epstein-Barr virus nuclear antigen 3C stabilizes Gemin3 to block p53-mediated apoptosis. PLoS Pathog..

[32] Stubenrauch F., Lim H.B., Laimins L.A. (1998). Differential requirements for conserved E2 binding sites in the life cycle of oncogenic human papillomavirus type 31. J. Virol.

[33] Thomas J.T., Hubert W.G., Ruesch M.N., Laimins L.A. (1999). Human papillomavirus type 31 oncoproteins E6 and E7 are required for the maintenance of episomes during the viral life cycle in normal human keratinocytes. Proc. Natl. Acad. Sci. USA.

[34] Park R.B., Androphy E.J. (2002). Genetic analysis of high-risk e6 in episomal maintenance of human papillomavirus genomes in primary human keratinocytes. J. Virol..

[35] De Geest K., Turyk M.E., Hosken M.I., Hudson J.B., Laimins L.A., Wilbanks G.D. (1993). Growth and differentiation of human papillomavirus type 31b positive human cervical cell lines. Gynecol Oncol.

[36] Stanley M. (1998). The immunology of genital human papilloma virus infection. Eur. J. Dermatol..

[37] Doorbar J. (2005). The papillomavirus life cycle. J. Clin. Virol..

[38] Munger K., Basile J.R., Duensing S., Eichten A., Gonzalez S.L., Grace M., Zacny V.L. (2001). Biological activities and molecular targets of the human papillomavirus E7 oncoprotein. Oncogene.

[39] McBride A.A., Oliveira J.G., McPhillips M.G. (2006). Partitioning viral genomes in mitosis: Same idea, different targets. Cell. Cycle.

[40] Peh W.L., Brandsma J.L., Christensen N.D., Cladel N.M., Wu X., Doorbar J. (2004). The viral E4 protein is required for the completion of the cottontail rabbit papillomavirus productive cycle in vivo. J. Virol..

[41] Genther S.M., Sterling S., Duensing S., Munger K., Sattler C., Lambert P.F. (2003). Quantitative role of the human papillomavirus type 16 E5 gene during the productive stage of the viral life cycle. J. Virol.

[42] Fehrmann F., Laimins L.A. (2003). Human papillomaviruses: Targeting differentiating epithelial cells for malignant transformation. Oncogene.

[43] Hummel M., Hudson J.B., Laimins L.A. (1992). Differentiation-induced and constitutive transcription of human papillomavirus type 31b in cell lines containing viral episomes. J. Virol.

[44] Ozbun M.A., Meyers C. (1997). Characterization of late gene transcripts expressed during vegetative replication of human papillomavirus type 31b. J. Virol..

[45] Davy C., McIntosh P., Jackson D.J., Sorathia R., Miell M., Wang Q., Khan J., Soneji Y., Doorbar J. (2009). A novel interaction between the human papillomavirus type 16 E2 and E1--E4 proteins leads to stabilization of E2. Virology.

[46] McIntosh P.B., Laskey P., Sullivan K., Davy C., Wang Q., Jackson D.J., Griffin H.M., Doorbar J. (2010). E1--E4-mediated keratin phosphorylation and ubiquitylation: A mechanism for keratin depletion in HPV16-infected epithelium. J. Cell Sci..

[47] Venuti A., Paolini F., Nasir L., Corteggio A., Roperto S., Campo M.S., Borzacchiello G. (2011). Papillomavirus E5: The smallest oncoprotein with many functions. Mol. Cancer.

[48] Gandarillas A., Goldsmith L.A., Gschmeissner S., Leigh I.M., Watt F.M. (1999). Evidence that apoptosis and terminal differentiation of epidermal keratinocytes are distinct processes. Exp. Dermatol..

[49] Hanahan D., Weinberg R.A. (2000). The hallmarks of cancer. Cell.

[50] Giri I., Yaniv M. (1988). Structural and mutational analysis of E2 trans-activating proteins of papillomaviruses reveals three distinct functional domains. Embo. J..

[51] Desaintes C., Demeret C., Goyat S., Yaniv M., Thierry F. (1997). Expression of the papillomavirus E2 protein in HeLa cells leads to apoptosis. Embo. J..

[52] Demeret C., Garcia-Carranca A., Thierry F. (2003). Transcription-independent triggering of the extrinsic pathway of apoptosis by human papillomavirus 18 E2 protein. Oncogene.

[53] Thierry F., Demeret C. (2008). Direct activation of caspase 8 by the proapoptotic E2 protein of HPV18 independent of adaptor proteins. Cell Death Differ..

[54] Massimi P., Pim D., Bertoli C., Bouvard V., Banks L. (1999). Interaction between the HPV-16 E2 transcriptional activator and p53. Oncogene.

[55] Webster K., Parish J., Pandya M., Stern P.L., Clarke A.R., Gaston K. (2000). The human papillomavirus (HPV) 16 E2 protein induces apoptosis in the absence of other HPV proteins and via a p53-dependent pathway. J. Biol. Chem..

[56] Green K.L., Brown C., Roeder G.E., Southgate T.D., Gaston K. (2007). A cancer cell-specific inducer of apoptosis. Hum. Gene Ther..

[57] Straight S.W., Hinkle P.M., Jewers R.J., McCance D.J. (1993). The E5 oncoprotein of human papillomavirus type 16 transforms fibroblasts and effects the downregulation of the epidermal growth factor receptor in keratinocytes. J. Virol..

[58] Genther Williams S.M., Disbrow G.L., Schlegel R., Lee D., Threadgill D.W., Lambert P.F. (2005). Requirement of epidermal growth factor receptor for hyperplasia induced by E5, a high-risk human papillomavirus oncogene. Cancer Res..

[59] Zhang B., Srirangam A., Potter D.A., Roman A. (2005). HPV16 E5 protein disrupts the c-Cbl-EGFR interaction and EGFR ubiquitination in human foreskin keratinocytes. Oncogene.

[60] Suprynowicz F.A., Krawczyk E., Hebert J.D., Sudarshan S.R., Simic V., Kamonjoh C.M., Schlegel R. (2010). The human papillomavirus type 16 E5 oncoprotein inhibits epidermal growth factor trafficking independently of endosome acidification. J. Virol..

[61] Dannenberg A.J., Lippman S.M., Mann J.R., Subbaramaiah K., DuBois R.N. (2005). Cyclooxygenase-2 and epidermal growth factor receptor: Pharmacologic targets for chemoprevention. J. Clin Oncol.

[62] Kabsch K., Alonso A. (2002). The human papillomavirus type 16 E5 protein impairs TRAIL- and FasL-mediated apoptosis in HaCaT cells by different mechanisms. J. Virol.

[63] Zhang B., Spandau D.F., Roman A. (2002). E5 protein of human papillomavirus type 16 protects human foreskin keratinocytes from UV B-irradiation-induced apoptosis. J. Virol.

[64] Oh J.M., Kim S.H., Cho E.A., Song Y.S., Kim W.H., Juhnn Y.S. (2010). Human papillomavirus type 16 E5 protein inhibits hydrogen-peroxide-induced apoptosis by stimulating ubiquitin-proteasome-mediated degradation of Bax in human cervical cancer cells. Carcinogenesis.

[65] Kim M.K., Kim H.S., Kim S.H., Oh J.M., Han J.Y., Lim J.M., Juhnn Y.S., Song Y.S. (2010). Human papillomavirus type 16 E5 oncoprotein as a new target for cervical cancer treatment. Biochem. Pharmacol..

[66] Scheffner M., Werness B.A., Huibregtse J.M., Levine A.J., Howley P.M. (1990). The E6 oncoprotein encoded by human papillomavirus types 16 and 18 promotes the degradation of p53. Cell..

[67] Huibregtse J.M., Scheffner M., Howley P.M. (1991). A cellular protein mediates association of p53 with the E6 oncoprotein of human papillomavirus types 16 or 18. Embo. J..

[68] Crook T., Tidy J.A., Vousden K.H. (1991). Degradation of p53 can be targeted by HPV E6 sequences distinct from those required for p53 binding and trans-activation. Cell..

[69] Lechner M.S., Laimins L.A. (1994). Inhibition of p53 DNA binding by human papillomavirus E6 proteins. J. Virol..

[70] Massimi P., Shai A., Lambert P., Banks L. (2008). HPV E6 degradation of p53 and PDZ containing substrates in an E6AP null background. Oncogene.

[71] Shai A., Pitot H.C., Lambert P.F. (2010). E6-associated protein is required for human papillomavirus type 16 E6 to cause cervical cancer in mice. Cancer Res..

[72] Thomas M., Banks L. (1998). Inhibition of Bak-induced apoptosis by HPV-18 E6. Oncogene.

[73] Gross-Mesilaty S., Reinstein E., Bercovich B., Tobias K.E., Schwartz A.L., Kahana C., Ciechanover A. (1998). Basal and human papillomavirus E6 oncoprotein-induced degradation of Myc proteins by the ubiquitin pathway. Proc. Natl. Acad. Sci. USA.

[74] Filippova M., Brown-Bryan T.A., Casiano C.A., Duerksen-Hughes P.J. (2005). The human papillomavirus 16 E6 protein can either protect or further sensitize cells to TNF: Effect of dose. Cell. Death Differ..

[75] Filippova M., Parkhurst L., Duerksen-Hughes P.J. (2004). The human papillomavirus 16 E6 protein binds to Fas-associated death domain and protects cells from Fas-triggered apoptosis. J. Biol. Chem..

[76] Filippova M., Filippov V.A., Kagoda M., Garnett T., Fodor N., Duerksen-Hughes P.J. (2009). Complexes of human papillomavirus type 16 E6 proteins form pseudo-death-inducing signaling complex structures during tumor necrosis factor-mediated apoptosis. J. Virol.

[77] Garnett T.O., Filippova M., Duerksen-Hughes P.J. (2006). Accelerated degradation of FADD and procaspase 8 in cells expressing human papilloma virus 16 E6 impairs TRAIL-mediated apoptosis. Cell. Death Differ..

[78] Du J., Chen G.G., Vlantis A.C., Chan P.K., Tsang R.K., van Hasselt C.A. (2004). Resistance to apoptosis of HPV 16-infected laryngeal cancer cells is associated with decreased Bak and increased Bcl-2 expression. Cancer Lett..

[79] Tungteakkhun S.S., Filippova M., Fodor N., Duerksen-Hughes P.J. (2010). The full-length isoform of human papillomavirus 16 E6 and its splice variant E6* bind to different sites on the procaspase 8 death effector domain. J. Virol..

[80] Filippova M., Song H., Connolly J.L., Dermody T.S., Duerksen-Hughes P.J. (2002). The human papillomavirus 16 E6 protein binds to tumor necrosis factor (TNF) R1 and protects cells from TNF-induced apoptosis. J. Biol. Chem..

[81] Borbely A.A., Murvai M., Konya J., Beck Z., Gergely L., Li F., Veress G. (2006). Effects of human papillomavirus type 16 oncoproteins on survivin gene expression. J. Gen. Virol..

[82] Jabbar S.F., Park S., Schweizer J., Berard-Bergery M., Pitot H.C., Lee D., Lambert P.F. (2012). Cervical cancers require the continuous expression of the human papillomavirus type 16 E7 oncoprotein even in the presence of the viral E6 oncoprotein. Cancer Res..

[83] Smotkin D., Prokoph H., Wettstein F.O. (1989). Oncogenic and nononcogenic human genital papillomaviruses generate the E7 mRNA by different mechanisms. J. Virol.

[84] Stacey S.N., Jordan D., Snijders P.J., Mackett M., Walboomers J.M., Arrand J.R. (1995). Translation of the human papillomavirus type 16 E7 oncoprotein from bicistronic mRNA is independent of splicing events within the E6 open reading frame. J. Virol..

[85] Pim D., Tomaic V., Banks L. (2009). The human papillomavirus (HPV) E6* proteins from high-risk, mucosal HPVs can direct degradation of cellular proteins in the absence of full-length E6 protein. J. Virol..

[86] Mesplede T., Gagnon D., Bergeron-Labrecque F., Azar I., Senechal H., Coutlee F., Archambault J. (2012). p53 degradation activity, expression, and subcellular localization of E6 proteins from 29 human papillomavirus genotypes. J. Virol.

[87] Filippova M., Johnson M.M., Bautista M., Filippov V., Fodor N., Tungteakkhun S.S., Williams K., Duerksen-Hughes P.J. (2007). The large and small isoforms of human papillomavirus type 16 E6 bind to and differentially affect procaspase 8 stability and activity. J. Virol.

[88] Münger K., Basile J., Duensing S., Eichten A., Gonzalez S., Grace M., Zacny L. (2001). Biological activities and molecular targets of the human papillomavirus E7 oncoprotein. Oncogene.

[89] Berezutskaya E., Yu B., Morozov A., Raychaudhuri P., Bagchi S. (1997). Differential regulation of the pocket domains of the retinoblastoma family proteins by the HPV16 E7 oncoprotein. Cell Growth Differ..

[90] Howes K.A., Ransom N., Papermaster D.S., Lasudry J.G., Albert D.M., Windle J.J. (1994). Apoptosis or retinoblastoma: Alternative fates of photoreceptors expressing the HPV-16 E7 gene in the presence or absence of p53. Genes Dev..

[91] Pan H., Griep A.E. (1995). Temporally distinct patterns of p53-dependent and p53-independent apoptosis during mouse lens development. Genes Dev..

[92] Pan H., Griep A.E. (1994). Altered cell cycle regulation in the lens of HPV-16 E6 or E7 transgenic mice: implications for tumor suppressor gene function in development. Genes Dev..

[93] Alunni-Fabbroni M., Littlewood T., Deleu L., Caldeira S., Giarre M., Dell' Orco M., Tommasino M. (2000). Induction of S phase and apoptosis by the human papillomavirus type 16 E7 protein are separable events in immortalized rodent fibroblasts. Oncogene.

[94] Kaznelson D.W., Bruun S., Monrad A., Gjerlov S., Birk J., Ropke C., Norrild B. (2004). Simultaneous human papilloma virus type 16 E7 and cdk inhibitor p21 expression induces apoptosis and cathepsin B activation. Virology.

[95] Stoppler H., Stoppler M.C., Johnson E., Simbulan-Rosenthal C.M., Smulson M.E., Iyer S., Rosenthal D.S., Schlegel R. (1998). The E7 protein of human papillomavirus type 16 sensitizes primary human keratinocytes to apoptosis. Oncogene.

[96] Thomas M., Glaunsinger B., Pim D., Javier R., Banks L. (2001). HPV E6 and MAGUK protein interactions: determination of the molecular basis for specific protein recognition and degradation. Oncogene.

[97] Magal S.S., Jackman A., Pei X.F., Schlegel R., Sherman L. (1998). Induction of apoptosis in human keratinocytes containing mutated p53 alleles and its inhibition by both the E6 and E7 oncoproteins. Int. J. Cancer.

[98] Severino A., Abbruzzese C., Manente L., Valderas A.A., Mattarocci S., Federico A., Starace G., Chersi A., Mileo A.M., Paggi M.G. (2007). Human papillomavirus-16 E7 interacts with Siva-1 and modulates apoptosis in HaCaT human immortalized keratinocytes. J. Cell. Physiol..

[99] Aguilar-Lemarroy A., Gariglio P., Whitaker N.J., Eichhorst S.T., zur Hausen H., Krammer P.H., Rosl F. (2002). Restoration of p53 expression sensitizes human papillomavirus type 16 immortalized human keratinocytes to CD95-mediated apoptosis. Oncogene.

[100] Kim C.Y., Tsai M.H., Osmanian C., Graeber T.G., Lee J.E., Giffard R.G., DiPaolo J.A., Peehl D.M., Giaccia A.J. (1997). Selection of human cervical epithelial cells that possess reduced apoptotic potential to low-oxygen conditions. Cancer Res..

[101] Brink A.A., Zielinski G.D., Steenbergen R.D., Snijders P.J., Meijer C.J. (2005). Clinical relevance of human papillomavirus testing in cytopathology. Cytopathology.

[102] Longworth M.S., Laimins L.A. (2004). Pathogenesis of human papillomaviruses in differentiating epithelia. Microbiol. Mol. Biol. Rev..

[103] Parkin D.M. (2006). The global health burden of infection-associated cancers in the year 2002. Int. J. Cancer.

[104] Widdice L.E.  (2012). Human papillomavirus disease in adolescents: Management and prevention. Adolesc Med. State Art Rev..

[105] Paavonen J., Jenkins D., Bosch F.X., Naud P., Salmeron J., Wheeler C.M., Chow S.N., Apter D.L., Kitchener H.C., Castellsague X. (2007). Efficacy of a prophylactic adjuvanted bivalent L1 virus-like-particle vaccine against infection with human papillomavirus types 16 and 18 in young women: An interim analysis of a phase III double-blind, randomised controlled trial. Lancet.

[106] Oehler-Janne C., Huguet F., Provencher S., Seifert B., Negretti L., Riener M.O., Bonet M., Allal A.S., Ciernik I.F. (2008). HIV-specific differences in outcome of squamous cell carcinoma of the anal canal: a multicentric cohort study of HIV-positive patients receiving highly active antiretroviral therapy. J. Clin. Oncol..

[107] Hagensee M.E., Cameron J.E., Leigh J.E., Clark R.A. (2004). Human papillomavirus infection and disease in HIV-infected individuals. Am. J. Med. Sci.

[108] Beutner K.R., Ferenczy A. (1997). Therapeutic approaches to genital warts. Am. J. Med..

[109] Beutner K.R., Tyring S.K., Trofatter K.F., Douglas J.M., Spruance S., Owens M.L., Fox T.L., Hougham A.J., Schmitt K.A. (1998). Imiquimod, a patient-applied immune-response modifier for treatment of external genital warts. Antimicrob Agents Chemother.

[110] Scheinfeld N., Lehman D.S. (2006). An evidence-based review of medical and surgical treatments of genital warts. Dermatol. Online J..

[111] D'Abramo C.M., Archambault J. (2011). Small molecule inhibitors of human papillomavirus protein-protein interactions. Open Virol. J..

[112] Phelps W.C., Barnes J.A., Lobe D.C. (1998). Molecular targets for human papillomaviruses: Prospects for antiviral therapy. Antivir. Chem. Chemother..

[113] Faucher A.M., White P.W., Brochu C., Grand-Maitre C., Rancourt J., Fazal G. (2004). Discovery of small-molecule inhibitors of the ATPase activity of human papillomavirus E1 helicase. J. Med. Chem..

[114] Hebner C., Beglin M., Laimins L.A. (2007). Human papillomavirus E6 proteins mediate resistance to interferon-induced growth arrest through inhibition of p53 acetylation. J. Virol..

[115] Scheffner M., Whitaker N.J. (2003). Human papillomavirus-induced carcinogenesis and the ubiquitin-proteasome system. Semin Cancer Biol..

[116] Thomas M.C., Chiang C.M. (2005). E6 oncoprotein represses p53-dependent gene activation via inhibition of protein acetylation independently of inducing p53 degradation. Mol. Cell..

[117] Baleja J.D., Cherry J.J., Liu Z., Gao H., Nicklaus M.C., Voigt J.H., Chen J.J., Androphy E.J. (2006). Identification of inhibitors to papillomavirus type 16 E6 protein based on three-dimensional structures of interacting proteins. Antiviral Res..

[118] Bellail A.C., Qi L., Mulligan P., Chhabra V., Hao C. (2009). TRAIL agonists on clinical trials for cancer therapy: the promises and the challenges. Rev. Recent Clin. Trials.

[119] El-Zawahry A., McKillop J., Voelkel-Johnson C. (2005). Doxorubicin increases the effectiveness of Apo2L/TRAIL for tumor growth inhibition of prostate cancer xenografts. BMC Cancer.

[120] Mom C.H., Verweij J., Oldenhuis C.N., Gietema J.A., Fox N.L., Miceli R., Eskens F.A., Loos W.J., de Vries E.G., Sleijfer S. (2009). Mapatumumab, a fully human agonistic monoclonal antibody that targets TRAIL-R1, in combination with gemcitabine and cisplatin: a phase I stud. Clin. Cancer Res..

[121] Naka T., Sugamura K., Hylander B.L., Widmer M.B., Rustum Y.M., Repasky E.A. (2002). Effects of tumor necrosis factor-related apoptosis-inducing ligand alone and in combination with chemotherapeutic agents on patients' colon tumors grown in SCID mice. Cancer Res..

[122] Yi C., Maksimoska J., Marmorstein R., Kissil J.L. (2010). Development of small-molecule inhibitors of the group I p21-activated kinases, emerging therapeutic targets in cancer. Biochem. Pharmacol..

[123] Yuan C.H., Filippova M., Tungteakkhun S.S., Duerksen-Hughes P.J., Krstenansky J.L. (2012). Small molecule inhibitors of the HPV16-E6 interaction with caspase 8. Bioorg. Med. Chem. Lett..

